# Superior effect of MP‐AzeFlu compared to monotherapy with fluticasone propionate or azelastine on GILZ, MKP‐1 and TTP anti‐inflammatory gene expression in healthy and inflamed upper airway mucosa

**DOI:** 10.1111/cea.14099

**Published:** 2022-02-04

**Authors:** Sònia Vicens‐Artés, Jordi Roca‐Ferrer, Valeria Tubita, Mireya Fuentes, Isam Alobid, Antonio Valero, Ferdinand Kopietz, DucTung Nguyen, Joaquim Mullol

**Affiliations:** ^1^ Clinical and Experimental Respiratory Immunoallergy IDIBAPS Barcelona Spain; ^2^ CIBER of Respiratory Diseases (CIBERES) Barcelona Spain; ^3^ Universitat de Barcelona Barcelona Spain; ^4^ Rhinology Unit & Smell Clinic ENT Department Hospital Clinic Barcelona Barcelona Spain; ^5^ Allergy Section Pulmonology & Allergy Department Barcelona Spain; ^6^ MEDA Pharma GmbH & Co. KG (A Viatris Company) Bad Homburg Germany

**Keywords:** azelastine, fluticasone, gene expression, GILZ, MKP‐1, MP‐AzeFlu, TTP

**Key Messages**

- MP‐AzeFlu has demonstrated efficacy in allergic rhinitis, and a superior effect compared with FP and AZE administered in monotherapy.
- We analysed the anti‐inflammatory effect of MP‐AzeFlu, compared with FP or AZE, on GILZ, MKP‐1 and TTP gene expression in healthy and inflamed upper airway mucosa.
- The superior clinical effect of MP‐AzeFlu compared to FP and AZE monotherapy may be related to greater up‐regulation of anti‐inflammatory GILZ gene expression and, to a lesser extent, MKP‐1 and TTP.

To the Editor,

Allergic rhinitis (AR) is a Th2 IgE‐mediated disease with elevated levels of pro‐inflammatory mediators, eosinophil infiltration of the nasal mucosa, and mucus hypersecretion. Antihistamines and intranasal corticosteroids, including MP‐AzeFlu (intranasal fluticasone and azelastine), are recommended as the first‐line therapy for AR.^1^ Intranasal corticosteroids are also the first‐line treatment for chronic rhinosinusitis with nasal polyps (CRSwNP) or without (CRSsNP), while antihistamines are only indicated in chronic rhinosinusitis (CRS) with concomitant AR.^2^

MP‐AzeFlu has demonstrated efficacy in AR, and a superior effect compared to these drugs administered individually, on nasal and ocular symptoms and quality of life; moreover, MP‐AzeFlu has shown earlier and faster AR control in moderate‐to‐severe AR.^3^ While MP‐AzeFlu is guideline‐recommended and its clinical efficacy has been widely studied, the mechanisms by which this therapy exerts its effect in AR are largely unknown.

Our laboratory has used an *in vitro* model of eosinophilic inflammation based on the interaction between cultured primary isolated nasal mucosa epithelial cells and isolated peripheral blood eosinophils to study the effect and potency of anti‐inflammatory drugs.^4^, ^5^ Using this model, we have previously reported greater anti‐inflammatory effects of the combination of an intranasal corticosteroid and an antihistamine, including MP‐AzeFlu, in both nasal mucosa and nasal polyp epithelial cells, compared to the effect of these drugs administered alone.^5^ We have also demonstrated that glucocorticoids induce the transcription of anti‐inflammatory genes such as glucocorticoid‐induced leucine zipper (GILZ), mitogen‐activated protein kinase (MAPK) phosphatase 1 (MKP‐1) and tristetraprolin (TTP) in nasal mucosa fibroblasts.^6^

While detailed insights are lacking regarding the mechanisms of pathogenesis in CRSwNP, it is now clear that IgE‐mediated Th2 inflammatory pathways play a critical role in this disease.^7^ H_1_ receptor antagonists decrease eosinophil survival^4^ and inhibit the release of pro‐inflammatory mediators by mast cells^8^ and the production of pro‐inflammatory cytokines by nasal epithelial cells.^4^, ^9^ In addition, azelastine (AZE) has been shown to enhance the anti‐inflammatory effect of budesonide and improve nasal symptoms in AR patients through the induction of MKP‐1 expression.^9^ As such, it is reasonable to hypothesize that an increased induction of anti‐inflammatory genes could partially explain the superior anti‐inflammatory effect of MP‐AzeFlu when compared to fluticasone propionate (FP) or AZE alone.

In the present study, we propose to analyse the anti‐inflammatory effect of MP‐AzeFlu, compared to monotherapy with FP or AZE, on GILZ, MKP‐1 and TTP gene expression in healthy and inflamed upper airway mucosa.

## 1 MATERIALS AND METHODS

### 1.1 Study population

Nasal mucosa (NM) tissues were obtained from patients undergoing nasal‐corrective surgery for nasal deviation and/or turbinate hypertrophy. Patients with current upper respiratory tract infections were excluded. Nasal polyps (NP) were obtained from patients undergoing nasal polypectomy and/or endoscopic sinus surgery for CRSwNP. Patients with asthma, nonsteroidal anti‐inflammatory drug‐exacerbated respiratory disease (N‐ERD) or other nasal and nasosinusal diseases (i.e. vasculitis, granulomatosis, benign or malignant tumours) were excluded.

### 1.2 Experimental design

Fibroblasts were isolated using a specific and selective growth culture medium. The purity of fibroblast cultures was confirmed by positive immunostaining to vimentin (fibroblast marker) and negative to cytokeratin 1 (epithelial cell marker).^6^ Cells were incubated with serum‐free medium and treated for 2–24 h with MP‐AzeFlu (dilution 1:10^2^–1:10^4^) or equivalent dilutions of FP and AZE. mRNA and protein expression of GILZ, MKP‐1 and TTP (at 2, 6 and/or 24 h) were assessed by real‐time polymerase chain reaction (RT‐PCR) and western blot, respectively.

### 1.3 Ethics issues

Prior to study initiation, the protocol was approved by the HCB Ethics Committee. Written informed consent was obtained from all patients prior to their participation.

### 1.4 Statistical methods

All results are expressed as mean ± standard error of the mean (SEM). The Wilcoxon tests were used for the non‐parametric paired comparisons. Analyses were performed using GraphPad 8.4.0 with an alpha level set at *p* < .05.

## 2 RESULTS

### 2.1 GILZ

**FIGURE 1:**
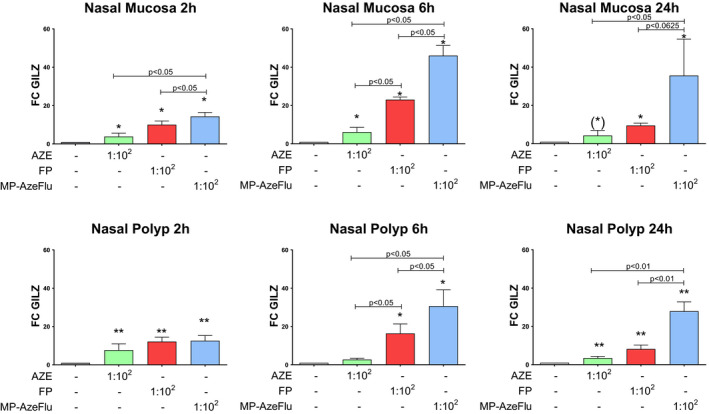
Superior effect of MP‐AzeFlu compared to monotherapy with fluticasone propionate (FP) or azelastine (AZE) on GILZ mRNA expression in nasal mucosa (NM) and nasal polyps (NP) in a time course (2, 6 and 24 h). *p < .05; **p < .01; (*)*p* = .06 compared to negative control

MP‐AzeFlu and FP up‐regulated GILZ mRNA expression at all time‐points (from 2 h to 24 h) and dilutions in both NM and NP. AZE induced a mild up‐regulation of GILZ mRNA expression at dilution 1:10^2^ at some time‐points. MP‐AzeFlu at dilution 1:10^2^ showed a superior effect in the up‐regulation of GILZ mRNA expression at 2 and 6 h in NM and at 6 and 24 h in NP compared to monotherapy with FP or AZE (Figure 1). Neither MP‐AzeFlu nor FP significantly up‐regulated GILZ protein expression at any time‐point or dilution.

### 2.2 MKP‐1

MP‐AzeFlu and FP up‐regulated MKP‐1 mRNA expression at all time‐points and dilutions in both NM and NP. AZE at dilution 1:10^2^ up‐regulated MKP‐1 mRNA expression at 2 h in both NM and NP. MP‐AzeFlu at dilution 1:10^2^ showed a partial superior effect in the up‐regulation of MKP‐1 mRNA expression compared to monotherapy with FP in NP (at 2 h) and AZE in NM (at 2 h and 6 h; Table 1). MKP‐1 protein expression was not detected when cells were incubated with MP‐AzeFlu and FP.

**TABLE 1:** Superior effect of MP‐AzeFlu compared with fluticasone propionate and azelastine monotherapy on MKP‐1 and TTP mRNA expression (2 and 6 h)

		MKP‐1 mRNA		TTP mRNA	
		NM	NP	NM	NP
2 h	MP‐AzeFlu	5.9 ± 1.1^†^	5.6 ± 1.4*	1.7 ± 0.1*^,†^	1.9 ± 0.3
	FP	4.5 ± 0.8	4.1 ± 1.0	1.3 ± 0.1	1.6 ± 0.4
	AZE	2.2 ± 0.4	4.7 ± 1.6	1.3 ± 0.2	1.4 ± 0.3
6 h	MP‐AzeFlu	6.9 ± 2.1^‡^	7.7 ± 3.2	1.8 ± 0.1^‡^	2.9 ± 0.2^†^
	FP	5.3 ± 0.8	6.4 ± 2.6	1.6 ± 0.1	2.1 ± 0.3
	AZE	1.6 ± 0.5	3.0 ± 1.2	1.0 ± 0.1	1.0 ± 0.1

Note

Data expressed as fold change (mean ± SEM) from untreated cells (control = 1).

Abbreviations: AZE, azelastine; FP, fluticasone propionate; NM, nasal mucosa; NP, nasal polyps.

*p < .05 compared to *FP*; ^†^ *p *< .05 compared to AZE; ^‡^ *p *< .01 compared to AZE.

### 2.3 TTP

MP‐AzeFlu and FP at dilution 1:10^2^ increased TTP mRNA expression in NM (at 2 h and 6 h) and NP (at 6 h).

AZE had no effect on TTP mRNA expression. MP‐AzeFlu at dilution 1:10^2^ showed a superior effect in the up‐regulation of TTP mRNA expression compared to monotherapy with FP and AZE in NM (at 2 h) and with AZE in NM and NP (at 6 h; Table 1). The effect of drugs on TTP protein expression was not studied due to very low gene expression.

## 3 DISCUSSION

In this study, MP‐AzeFlu, FP and AZE up‐regulated GILZ mRNA expression, with MP‐AzeFlu showing a superior effect compared with that of monotherapy. MP‐AzeFlu, FP and AZE also up‐regulated MKP‐1 mRNA expression; however, no significant differences were observed between MP‐AzeFlu and monotherapy. MP‐AzeFlu and FP up‐regulated TTP mRNA expression, while AZE did not.

Anti‐inflammatory genes are activated when glucocorticoids (GCs) diffuse across the cell membrane to bind to GRα. The ligand‐bound receptor translocates to the nucleus and binds GC‐responsive elements (GREs) on the promoter region of target genes.^10^ The anti‐inflammatory effect of MP‐AzeFlu has been confirmed by the up‐regulation of anti‐inflammatory gene expression of GILZ, MKP‐1 and TTP in our *in vitro* model. These findings suggest some of the molecular mechanisms of action of MP‐AzeFlu in the upper airway inflammation (both AR and CRSwNP). However, in vitro models have limitations. In this artificial setting, interactions between cells are lost. Furthermore, the concentration of drugs used do not correlate with clinical doses used in the patient care setting. Thus, these findings may not translate to clinical outcomes and further studies are needed to confirm the findings.

In conclusion, the superior clinical effect of MP‐AzeFlu on both NP and NM compared with monotherapy may be partly related to the greater up‐regulation of the anti‐inflammatory gene expression of GILZ and, to a lesser extent, MKP‐1 and TTP expression.

## CONFLICT OF INTEREST

Joaquim Mullol is or has been a member of national and international scientific advisory boards (consulting), received fees for lectures, and grants for research projects from Allakos, AstraZeneca, Genentech‐Roche, Glenmark, GSK, Menarini, MSD, Mitsubishi‐Tanabe, MYLAN‐MEDA Pharma (Viatris), Novartis, Procter & Gamble, Sanofi‐Genzyme & Regeneron, UCB and Uriach Group. SVA, JRF, VT, MF, IA and AV do not have conflicts of interest for the present manuscript.

## AUTHOR CONTRIBUTIONS

All authors made substantial contributions to the conception or design of the manuscript, or the acquisition, analysis or interpretation of data for the manuscript, and all authors were involved in drafting the manuscript or revising it critically for important intellectual content. The authors were fully responsible for all content and editorial decisions and received no financial support or other forms of compensation related to the development of this manuscript. All authors had final approval of the manuscript and are accountable for all aspects of the work in ensuring the accuracy and integrity of this manuscript.

## ETHICAL APPROVAL

The Dymecos 2 study was approved by the Ethics Committee (CEIm) from Hospital Clinic Barcelona (Catalonia, Spain) on 3 February 2016 with the Registration No. HCB/2016/0007.

## Data Availability

The data that support the findings of this study are available from the corresponding author upon reasonable request.
